# A Comparison of Whipple Outcomes Between a Safety-Net Hospital and American College of Surgeons National Surgical Quality Improvement Program (ACS-NSQIP) in African Americans

**DOI:** 10.7759/cureus.43487

**Published:** 2023-08-14

**Authors:** Keouna Pather, Erin M Mobley, Haytham H Alabbas, Ziad Awad

**Affiliations:** 1 Surgery, University of Florida College of Medicine – Jacksonville, Jacksonville, USA; 2 Surgery/General and Oncology Surgery, King Faisal Specialist Hospital and Research Center, Jeddah, SAU

**Keywords:** safety-net hospital, 30-day outcomes, black/african american, american college of surgeons national surgical quality improvement program, pancreaticoduodenectomy procedure

## Abstract

Purpose

The aim of this study was to compare 30-day adverse events following pancreaticoduodenectomy between our safety-net hospital and the American College of Surgeons National Surgical Quality Improvement Program (ACS-NSQIP) in a matched cohort of Black/African American (AA) patients.

Methods

We retrospectively reviewed consecutive Black/AA patients undergoing pancreaticoduodenectomies from 2015 to 2020 at our safety-net institution. The corresponding patients from the ACS-NSQIP (2015-2019) were queried. Propensity-score matching was performed between safety-net and ACS-NSQIP Black/AA cohorts to equate baseline characteristics, and 30-day outcomes were compared between propensity-matched cohorts.

Results

Thirty-two Black/AAs (16 females; 62.1±10.7 years) were identified from 128 patients undergoing pancreaticoduodenectomies at our safety-net institution and were propensity-score matched to 32 ACS-NSQIP patients. After matching, baseline characteristics did not significantly differ between cohorts. Postoperatively, surgical site infections, wound disruptions, respiratory events, cardiovascular events, urinary tract infections, acute renal failure, sepsis, delayed gastric emptying, and pancreatic fistulas were not significantly different between our safety-net and ACS-NSQIP cohorts. Our length of stay (LOS) was longer (17.0(12.3-27.0) versus 10.0(7.0-16.0) days); however, patients with a LOS>30 days were comparable. Furthermore, 30-day readmissions were similar, and 30-day reoperations were lower (p=0.03) at our safety-net institution.

Conclusions

Black/AA patients who underwent pancreatectomies at a safety-net hospital had similar outcomes and fewer reoperations compared to a corresponding national cohort. Although we illustrate comparable outcomes, clinical pathways to mitigate and alleviate health disparities in marginalized populations at a safety-net hospital should be emphasized to continue improving outcomes.

## Introduction

Pancreatic resection is a highly complex surgical procedure that carries a consequential risk of morbidity; nonetheless, this procedure remains the mainstay of potentially curative treatment for pancreatic cancer. Surgical resection significantly improves disease-specific survival compared with patients without resection, especially for early-stage, non-metastatic pancreatic adenocarcinoma [1-4].

Efforts to improve surgical outcomes have developed over time [5]. However, historic and institutional racial inequities in cancer care have led to significant disparities [5,6]. Authors of previous studies comparing cancer-related outcomes in Black/African American (AA) and white patients report that on average Black/AA patients present with pancreatic cancer at younger ages, with later-stage disease, and are less likely to receive any treatment modality, including surgical resection or chemoradiation therapy [6-10]. Additionally, Black/AA patients are less likely to receive treatment at high-volume or academic institutions, have an increased risk of postoperative complications (such as sepsis, pneumonia, and acute kidney injury), have a longer length of stay (LOS), and a lower survival rate compared with white patients [7,9-12].

Our institution is an urban teaching center and the only level 1 trauma center in the region. Our institution also qualifies as a hospital with a high safety-net burden that serves an area of underinsured and uninsured patients in Northeast Florida and serves as the regional referral center for patients requiring complex cancer procedures. Notably, our patients are predominantly high-risk surgical candidates often harboring substantial comorbidities. Given the healthcare inequities that exist for Black/AA patients and the safety-net burden at our hospital, we sought to investigate differences in our Black/AA patient population compared with a national sample. Related to this, we are mindful that our Black/AA patient population differs in baseline characteristics from patients seen at other hospitals across the country, likely due to the complex and multifactorial interplay of serving as a regional referral center, with the high uninsured or underinsured patient mix, and a substantial number of patients with a high comorbidity burden. Consequently, a direct comparison of our patients to the rest of the country should be limited to peer safety-net institutions [13]. Motivated by this, we compared 30-day adverse events following pancreaticoduodenectomy between our safety-net hospital and a propensity score-matched population of Black/AA patients using data from the American College of Surgeons National Surgical Quality Improvement Program (ACS-NSQIP).

## Materials and methods

Study design and data source

This was a retrospective analysis of consecutive patients undergoing a non-pylorus-preserving pancreaticoduodenectomy (classic Whipple procedure) at the University of Florida Health-Jacksonville. The study was approved by our Institutional Review Board (IRB202002410). All consecutive patients treated with a non-emergent Whipple operation by the senior author (ZTA) from August 2015 to December 2020 were included. Baseline characteristics, pancreatic disease characteristics, procedural details, LOS, discharge destination, and 30-day postoperative adverse events were extracted from our institution’s medical record. The ACS-NSQIP targeted pancreatectomy participant use data files from 2015 to 2019 were merged into the associated main ACS-NSQIP dataset to collectively include pancreatectomy-specific, preoperative, procedural, and postoperative variables [14]. Black/AA adult patients undergoing classic Whipple procedures were selected, which included current procedural terminology codes 48150 and 48152.

 Preoperative baseline patient characteristics that were available from both our safety-net hospital and ACS-NSQIP data included patient demographics (age, sex, race) and preoperative medical conditions of body mass index (BMI), diabetes mellitus, hypertension, chronic obstructive pulmonary disease (COPD), presence of ascites, obstructive jaundice, current smoker, and American Society of Anesthesiologist category. Included pancreatic disease characteristics were the use of neoadjuvant chemoradiation within 90 days and pathological tumor, node, and metastases stage. Procedural details were elective surgery, surgical approach, unplanned conversion to open, and operative time. Postoperative variables included LOS, discharge destination to home (home or facility where the patient was living prior to admission), and 30-day postoperative adverse events.

Thirty-day adverse event outcomes

Thirty-day postoperative outcomes included superficial incisional surgical site infection (SSI), deep incisional SSI, organ/space SSI, wound disruption, cardiovascular events, respiratory events, urinary tract infections (UTIs), acute renal failure, sepsis, delayed gastric emptying, pancreatic fistula, and 30-day readmissions and reoperations. Cardiovascular events included stroke, cardiac arrest, myocardial infarction, and deep vein thrombosis. Respiratory events were pneumonia, unplanned intubation, and pulmonary embolism. Adverse events were collected using the ACS-NSQIP definitions [15]. Thirty-day outcomes were compared between a propensity score-matched cohort of Black/AA patients who underwent pancreaticoduodenectomy at our safety-net institution from 2015 to 2020 and the ACS-NSQIP from 2015 to 2019. The same time period was not included due to limitations in available data. Mortality was not compared between our safety-net hospital and ACS-NSQIP as 98% of data was missing from the ACS-NSQIP mortality variable.

Statistical analysis

Propensity score matching was used to compare outcomes in Black/AA patients among our safety-net hospital cohort and ACS-NSQIP cohort. The PSMATCH algorithm in SAS® was used to calculate propensity scores for each group, in which a multivariable logistic regression model based on patients’ baseline characteristics was performed to determine the likelihood of patients being in a safety-net hospital versus ACS-NSQIP group [16]. A greedy nearest neighbor matching model without replacement and a caliper width of 0.2 standard deviations was then used to perform a 1:1 match ratio of Black/AA patients from our safety-net hospital to Black/AA patients from the ACS-NSQIP. The final sample included the propensity-matched pairs.

 Bivariate analyses were used to compare patient baseline characteristics, procedural details, and postoperative 30-day adverse events between matched cohorts. Categorical and continuous variables were reported as frequencies and counts and as mean and standard deviation or median and interquartile range (IQR), respectively. McNemar’s and McNemar-Bowker’s tests were used to analyze dichotomous and nominal categorical variables, respectively. Analysis of parametric continuous variables was performed using a paired t-test, and ordinal categorical and non-parametric continuous variables were analyzed using a Wilcoxon signed-rank test. Statistical significance was defined by two-sided p-values of <0.05. All statistical analyses were conducted using SAS® University Edition 9.4 (SAS Institute Inc., Cary, NC, USA).

## Results

Baseline patient and disease characteristics

A total of 128 consecutive patients underwent a pancreaticoduodenectomy from August 2015 to December 2020 at our safety-net hospital, of which 32 were Black/AA patients (25%). Between 2015 and 2019, a total of 1,076 Black/AA patients underwent pancreaticoduodenectomy at participating ACS-NSQIP institutions (8%). Between the overall unmatched safety-net cohort and ACS-NSQIP cohort, baseline characteristics of BMI (p=0.01), particularly BMI > 30 (p<0.01), and N stage (p=0.02) were significantly different. Of note, after propensity score matching, there were no significant differences in baseline characteristics between safety-net (n=32, mean age 62.1±10.7 years old, 16 females) and ACS-NSQIP cohorts (n=32, mean age 60.6±11.7 years old, 12 females; Table 1).

**Table 1:** Baseline characteristics in an African American cohort matched between a safety-net hospital and the ACS-NSQIP ACS-NSQIP, American College of Surgeons National Surgical Quality Improvement Program; ASA, American Society of Anesthesiologists physical status score.

	Safety-Net Hospital, n=32	ACS-NSQIP, n=32	P-value
	n (percent, %) or mean ± standard deviation		
Demographics			
Age	62.1 ± 10.7	60.6 ± 11.7	0.59
Sex			
Female	16 (50)	12 (38)	0.42
Male	16 (50)	20 (63)	
Comorbidities			
Body Mass Index, BMI	31.4 ± 6.1	30.1 ± 5.8	0.38
BMI ≥ 30	19 (59)	16 (50)	0.55
Diabetes mellitus	15 (47)	16 (50)	1.00
Hypertension	25 (78)	20 (63)	0.30
Obstructive jaundice	11 (34)	10 (31)	1.00
Current smoker	3 (9)	2 (6)	1.00
Preoperative Risk Assessment			
ASA 1	0 (0)	0 (0)	0.72
ASA 2	4 (13)	7 (22)	
ASA 3	24 (75)	20 (63)	
ASA 4	4 (13)	5 (16)	
Disease characteristics			
Neoadjuvant chemotherapy	3 (9)	8 (25)	0.18
Neoadjuvant radiation therapy	0 (0)	2 (6)	0.50
Tumor (T) stage			
T0	0 (0)	1 (3)	0.15
T1	2 (6)	2 (6)	
T2	9 (28)	9 (28)	
T3	14 (44)	15 (47)	
T4	0 (0)	2 (6)	
Tis	0 (0)	1 (3)	
Tx	0 (0)	0 (0)	
N/A	7 (22)	2 (6)	
Node (N) stage			
N0	12 (38)	8 (25)	0.74
N1	9 (28)	17 (53)	
N2	6 (19)	5 (16)	
N3	0 (0)	0 (0)	
Nx	0 (0)	0 (0)	
N/A	5 (16)	2 (6)	

In Black/AA patients at our safety-net hospital, pancreatic ductal adenocarcinoma was the most commonly encountered pathology (n=15), followed by cholangiocarcinoma (n=4), neuroendocrine tumors (n=4), and other abnormalities (n=8). Lesions in Black/AA patients from the ACS-NSQIP cohort included pancreatic ductal adenocarcinoma (n=22), duodenal carcinoma (n=3), neuroendocrine tumors (n=2), and other pathology (n=4). Whipple procedures performed for chronic pancreatitis occurred in one patient in each cohort, respectively. There were no significant differences in lesion types among the cohorts (p=0.14).

Procedural details

 Elective surgery occurred in 26 (81%) and 25 (78%) Black/AA patients from our safety-net hospital and ACS-NSQIP cohorts, respectively (Table 2). Open surgery was the most common approach in both safety-net and ACS-NSQIP cohorts (28 patients versus 27 patients) and an unplanned conversion to open surgery occurred in three patients and one patient, respectively (p=0.63). Operative time was comparable between our safety-net hospital cohort and the ACS-NSQIP cohort (433.1±97.4 versus 389.8±148.3 minutes; p=0.13).

**Table 2:** Procedural details and postoperative events in a cohort of matched African American patients ACS-NSQIP, American College of Surgeons National Surgical Quality Improvement Program. *Statistically significant (p-value < 0.05).

	Safety-Net Hospital, n=32	ACS-NSQIP, n=32	P-value
	n (percent, %) or median [interquartile range] or mean ± standard deviation		
Procedural details			
Elective surgery	26 (81)	25 (78)	1.00
Surgical approach			
Open	28 (88)	27 (84)	1.00
Laparoscopic	2 (6)	0 (0)	
Robotic	2 (6)	5 (16)	
Hybrid	0 (0)	0 (0)	
Conversion to open	3 (9)	1 (3)	0.63
Operative time (minutes)	433.1 ± 97.4	389.8 ± 148.3	0.13
Length of stay and discharge destination			
Total length of stay (days)	17.0 [12.3-27.0]	10.0 [7.0-16.0]	<0.01*
Length of stay > 30 days	6 (19)	2 (6)	0.22
Discharge to home	21 (66)	24 (75)	0.29
30-day adverse events			
Superficial incisional surgical site infection, SSI	7 (22)	1 (3)	0.07
Deep incisional SSI	3 (9)	0 (0)	0.25
Organ/space SSI	11 (34)	5 (16)	0.21
Wound disruption	2 (6)	0 (0)	0.50
Cardiovascular events			
Stroke	0 (0)	0 (0)	
Cardiac arrest	1 (3)	0 (0)	1.00
Myocardial infarction	0 (0)	2 (6)	0.50
Deep vein thrombosis	1 (3)	0 (0)	1.00
Respiratory events			
Pneumonia	3 (9)	1 (3)	0.63
Unplanned reintubation	2 (6)	1 (3)	1.00
Pulmonary embolism	1 (3)	0 (0)	1.00
Urinary tract infection	7 (22)	1 (3)	0.07
Acute renal failure	1 (3)	1 (3)	1.00
Sepsis	8 (25)	3 (9)	0.18
Delayed gastric emptying	3 (9)	4 (13)	1.00
Pancreatic fistula	8 (25)	7 (22)	1.00
Readmissions	3 (9)	8 (25)	0.23
Reoperations	0 (0)	6 (19)	0.03*

Superficial incisional (p=0.07), deep incisional (p=0.25), organ/space (p=0.21) SSIs, and wound disruptions (p=0.50) were not significantly different between Black/AA patients from our safety-net and ACS-NSQIP cohorts (Table 2). Similarly, cardiovascular and respiratory events were comparable among cohorts. There was also no significant difference in UTIs (p=0.07), acute renal failure (p=1.00), sepsis (p=0.18), delayed gastric emptying (p=1.00), and pancreatic fistulas (p=1.00) between the cohorts. LOS was significantly higher in the safety-net cohort (17.0, IQR 12.3-27.0 days) compared with the ACS-NSQIP cohort (10.0, IQR 7.0-16.0 days; p<0.01); however, the number of patients with a LOS > 30 days did not significantly differ among the cohorts (p=0.22). The number of patients discharged to home was similar (p=0.29) and 30-day readmissions did not significantly differ between the cohorts (p=0.23). Notably, 30-day reoperations were significantly lower at our safety-net hospital compared with ACS-NSQIP (n=0 versus n=6; p=0.03).

## Discussion

Our propensity score-matched study of 30-day adverse events following pancreaticoduodenectomy from 2015 to 2020 suggests Black/AA patients at our safety-net hospital have similar cardiac, respiratory, wound, and infection-related outcomes compared to Black/AA patients in a corresponding national ACS-NSQIP sample. In contrast, Black/AA patients at our safety-net hospital have fewer reoperations, and a longer LOS compared to Black/AA patients in the same corresponding national ACS-NSQIP sample.

Hospitals with a high safety-net burden serving low-income and vulnerable patient populations have previously reported inferior surgical outcomes with increased costs as compared with non-safety-net hospitals [13,17,18]. However, these outcomes were not assessed at the individual institution level. Hoehn and colleagues reported an increased number of Black/AA and younger patients, coupled with higher rates of in-hospital mortality, 30-day readmissions, longer LOS, and higher costs for pancreaticoduodenectomy procedures at high safety-net burden hospitals compared with low-burden hospitals [13]. After adjusting for patient baseline characteristics and hospital volume, outcomes remained suboptimal; therefore, the authors suggest that intrinsic factors of safety-net hospitals lead to lower quality and higher cost of surgical care [13].

Our institution qualifies as a regional safety-net facility with a high proportion of Medicare and Medicaid-insured patients undergoing complex surgeries [19]. In contrast to prior reports, our study demonstrated that postoperative complications following pancreaticoduodenectomy procedures in Black/AA patients at our safety-net hospital were comparable to a matched national cohort [6,11,13,20]. Notably, 30-day unplanned reoperations were lower and 30-day readmissions were comparable to the national sample. Therefore, this suggests that a combination of preoperative comorbidity optimization, operative technique, postoperative care, and quality management were sufficient to manage complications and prevent reoperations and readmissions at our safety-net institution. These results contradict prior studies in which readmission rates were higher at safety-net hospitals and do not necessarily reflect the notion that intrinsic qualities of safety-net hospitals account for outcome discrepancies, at least at our institution [13,18,21].

LOS was longer at our institution compared with a national sample. Moreover, we would be remised to not underscore that postoperative complications do not solely justify this longer LOS as the complications in our group of patients were comparable to a national sample. Authors of previous studies have reported similar findings after cancer surgery and noncancer surgeries, in which perioperative outcomes were comparable among racial and ethnic minority groups and white patients; however, LOS was longer in Black patients [22,23]. Undoubtedly, variations in LOS are multifactorial, and delayed discharge is often associated with lower socioeconomic status, access to rehabilitation, insufficient insurance coverage, and patient-specific attributes, such as caregiver support. In addition to access to adequate rehabilitation services, early integration of these services and care coordination, well prior to discharge, will help assist with decreasing LOS and lower the cost of occupying a surgical bed in a busy unit. Pancreaticoduodenectomy is a complex surgery that requires coordination and collaboration between multiple resources, particularly for cancer patients. Given our safety-net environment and the higher proportion of underinsured patients, a potential limitation in resource availability may arise for this vulnerable population. This restriction may influence LOS as discharge disposition with access to adequate care for vulnerable patients may become challenging; therefore, increasing hospitalization. Moreover, this increased LOS in the presence of comparable postoperative complications underscores the need to further evaluate the complex interaction between race, baseline comorbidities, and social, cultural, and economic structures that impact health and access to care among vulnerable populations at our safety-net hospital. Overall, emphasis to mitigate and alleviate healthcare disparities in a marginalized population at a safety-net hospital should be continually addressed.

Limitations

The primary limitation of this study is the retrospective nature and small study sample which limits the statistical power needed to elucidate all clinically significant disparities. Furthermore, outcomes were limited to 30 days based on ACS-NSQIP availability with a substantial amount of missing for the 30-day mortality variable. Additionally, the lack of access to comprehensive clinical and demographic data available through ACS-NSQIP, such as pre- and post-operative performance status and socioeconomic factors, may have influenced our study findings. Given the distinct differences in a safety-net hospital’s patient population, propensity score matching analysis was used. Although this statistical method has limitations such as study sample size reduction, it is useful to allow comparison accounting for variations in preoperative covariates that impact postoperative outcomes.

## Conclusions

Black/AA patients who underwent pancreatectomies at our safety-net hospital had similar outcomes and fewer reoperations compared to a corresponding national sample. While these results suggest quality care is provided to Black/AA patients at our institution, clinical pathways to mitigate and alleviate health disparities in marginalized populations at a safety-net hospital should continually be emphasized to improve surgical outcomes.
